# An Enhanced Probabilistic LDA for Multi-Class Brain Computer Interface

**DOI:** 10.1371/journal.pone.0014634

**Published:** 2011-01-31

**Authors:** Peng Xu, Ping Yang, Xu Lei, Dezhong Yao

**Affiliations:** Key Laboratory for NeuroInformation of Ministry of Education, School of Life Science and Technology, University of Electronic Science and Technology of China, Chengdu, China; University of California Irvine, United States of America

## Abstract

**Background:**

There is a growing interest in the study of signal processing and machine learning methods, which may make the brain computer interface (BCI) a new communication channel. A variety of classification methods have been utilized to convert the brain information into control commands. However, most of the methods only produce uncalibrated values and uncertain results.

**Methodology/Principal Findings:**

In this study, we presented a probabilistic method “*enhanced BLDA*” (EBLDA) for multi-class motor imagery BCI, which utilized Bayesian linear discriminant analysis (BLDA) with probabilistic output to improve the classification performance. EBLDA builds a new classifier that enlarges training dataset by adding test samples with high probability. EBLDA is based on the hypothesis that unlabeled samples with high probability provide valuable information to enhance learning process and generate a classifier with refined decision boundaries. To investigate the performance of EBLDA, we first used carefully designed simulated datasets to study how EBLDA works. Then, we adopted a real BCI dataset for further evaluation. The current study shows that: 1) Probabilistic information can improve the performance of BCI for subjects with high kappa coefficient; 2) With supplementary training samples from the test samples of high probability, EBLDA is significantly better than BLDA in classification, especially for small training datasets, in which EBLDA can obtain a refined decision boundary by a shift of BLDA decision boundary with the support of the information from test samples.

**Conclusions/Significance:**

The proposed EBLDA could potentially reduce training effort. Therefore, it is valuable for us to realize an effective online BCI system, especially for multi-class BCI systems.

## Introduction

Brain computer interface (BCI) is a new communication channel that directly translates brain activities into control commands or messages for peripheral equipments. BCI may enable the disabled to control a computer application or a neuro-prosthesis [1], [2]. For both laboratory study and practical application, accuracy and information transfer rates (ITR) [3] are two important factors for BCI performance evaluation. At present, BCI applicability is severely limited by its unsatisfactory ITR and accuracy. A feasible way to increase ITR of a BCI system is to change the usual binary decision to a more diverse decision [4], [5].

However, when the number of brain patterns increases, both signal processing (feature extraction) and machine learning (pattern classification) will encounter difficulties. For example, the classification accuracy may decrease due to the interference of the new patterns. Currently, some classifiers (e.g., the linear discriminant analysis (LDA), multilayer perception, nearest neighbor classifier, and combined algorithms [6]) have been introduced for a multi-class BCI. However, most of the classifiers only use the information in training set without considering the possible change of the statistic properties between training and test sets.

Recently, certain probabilistic methods (e.g., Gaussian processes [7], Bayesian learning [8], [9]) have been introduced to improve the robustness and generalization of a BCI. In recent studies, because of its low computational complexity and immunity to overfit, LDA classifier is often preferred over its nonlinear counterparts in BCI, especially when a small number of samples are available for training [10]. Motivated by the success of LDA in BCI, Hoffman et al. developed an evidence framework based Bayesian LDA (BLDA), and verified its usefulness in a P300 based BCI [11]. Although probabilistic methods may provide confidence level of the output that is meaningful for further post-processing (e.g., classifiers combination [11],[12]), these algorithms have not been discussed in terms of solving multi-class problem in BCI.

By focusing on the application of BLDA in multi-class motor imagery task, this paper proposed an enhanced BLDA (EBLDA), which could increase the performance of BCI by using the information mined from test samples (i.e., adding reliable tested samples with high classification probability to the training set to further improve the classifier performance).

The paper is organized as follows. Section *Materials and Methods* provides a detailed description of BLDA and EBLDA, and the simulated dataset, the BCI experimental dataset, and the pre-processing techniques (e.g., selection of filters, time interval, and feature extraction) are included in this section too. Results are provided in Section *Results*. Section *Discussion* is a general discussion for the results.

## Materials and Methods

### 1. BLDA

BLDA is based on the evidence framework for Bayesian regression and has been certified very useful in a P300 based BCI [11]. BLDA is a Bayesian version of regularized LDA, in which regularization parameters are estimated with Bayesian regression. Previous studies have revealed that compared with LDA, BLDA is more competitive for the conditions with a small number of train sets or strong noise contamination [9], [11].

Assuming that the target 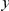 and feature vector 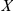 are linearly related, and bias *z* has Gaussian form, the linear classifier should have a form as follows,
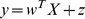
(1)Let 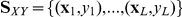 be the training set composed of feature vector 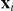 and the corresponding binary states 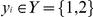. From (1) we can obtain the likelihood function for the weights 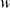 as,
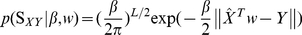
(2)Where 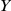 denotes a vector containing all the training targets, 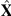 denotes the matrix that is obtained from the horizontal stacking of the training feature vectors, 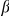 denotes the inverse variance of the noise, and *L* denotes the number of training samples. In BLDA, combing the bias *z* into the weights *w*, by expanding the dimension of *w*, i.e., the last entry of *w* is the bias term, the prior distribution of the weights 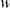 is assumed as,
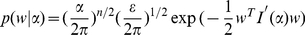
(3)where *w* is extended to *n+1* dimension with last entry being the bias term, and 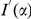 is a diagonal matrix with *n+1* dimension having form as
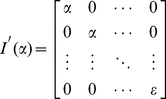
(4)The prior for the weights is thus an isotropic, zero-mean Gaussian distribution with variance 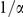, and the prior for the bias being the last entry in *w*, is a zero-mean univariate Gaussian process with variance 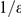, where 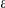 is a small bias value for overcoming the danger of overfitting. *n* is the dimension of a feature vector. From the likelihood function and prior distribution, the posterior distribution can be computed by using Bayes rule as,
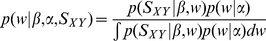
(5)


Since the prior distribution and likelihood distribution are Gaussian, the posterior distribution also has Gaussian form and the distribution can be determined by the mean (*m*) of w and covariance (*C* ) as follows,
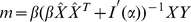
(6)

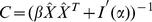
(7)


From the posterior distribution and likelihood function, the predictive distribution can be obtained by inserting a new test feature vector 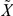 as,
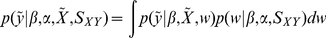
(8)where 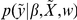 denotes a normal distribution. The predictive distribution can be characterized by its means (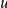) and variance (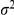) as,
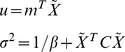
(9)Accordingly, we can calculate the probability of feature 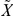 belonging to class label *y* = 1(similar to class label *y* = −1) as,
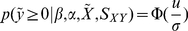
(10)where 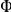 denotes the standard normal cumulative distribution function.

Obviously, the probabilistic output (10) depends on the mean 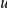 and variance 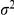 calculated from equation (9). Therefore, both the posterior distribution (5) of 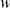 and the predictive distribution (10) of 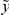 depend on the parameters 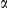 and 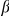. In BLDA, the parameter selection problem is solved efficiently by maximum-likelihood estimates [11]:
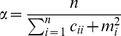
(11)

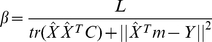
(12)where *n* denotes the dimension of a feature vector and *L* denotes the number of training samples. BLDA uses an iterative scheme to estimate the parameters as : 1) *C* and *m* are computed for an initial value of α and β; 2) the hyperparameters are updated according to (11) and (12); 3) Equations (6), (7), and (11), (12) are iterated to obtain the predictive distribution until the values for the hyperparameters converged; 4) the probability that feature 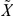 belongs to class 1 by standard normal cumulative distribution function is calculated by (9) and (10).

At last, the linear decision boundary for binary problems is,
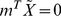
(13)


### 2. EBLDA

In order to release the training effort and improve the performance of a BCI system, this paper proposed a post-processing algorithm by rebuilding a new classifier through adding additional test samples with high probability to training sets. We refer to the new algorithm as enhanced Bayesian LDA (EBLDA). Generally, the size of training sets can influence the performance of classifiers in two ways. On one hand, a large training set may contain more outliers and artifacts, which may reduce classification accuracy for learning machine because the classifier may overfit the training data. On the other hand, a small training set may not provide enough information for classification. Therefore, the classification accuracy cannot be guaranteed either.

We supposed that unlabeled samples with high classification probability may provide valuable information to enhance the learning process and generate a classifier with refined decision boundaries. Hence, in our study, probabilistic information from BLDA was regarded as a confident evaluation criterion to select reliable test samples, which could enlarge the training set. For example, in a binary class problem that contained a positive and negative class, we obtained the probability from BLDA classifier for a test sample. When the probability of a sample was lager than a relatively strict threshold (such as 0.9), this test sample would be added to the training set for classifier calibration.

### 3. Simulation and real data tests

In this paper, based on the finding that that BLDA is more robust in the BCI application compared with other approaches like LDA, MD, SVM [9], [11], BLDA serves as the baseline for performance comparison.

### 3.1 The Simulated Data Sets

To explore when and why BLDA and EBLDA are effective in practice, we first constructed a simulated data set, whose exact decision boundaries were known. Based on this simulated dataset, we estimated the parameters of BLDA and EBLDA to obtain their corresponding decision boundaries.

The data set consists of two 2-dimensional normal distributions. The positive class (labeled *y* = 1) and the negative class (label *y* = −1) are given by below model parameters,
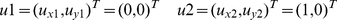


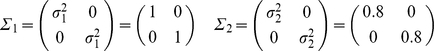
where 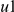 and 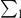 are the mean and covariance matrix for class labeled with 1, and 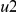 and 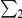 are the mean and covariance matrix for class labeled with −1.

Mathematically, the Bayesian optimal decision boundary of this data set is,
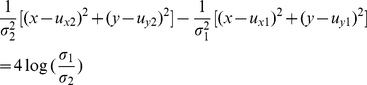
(14)


### 3.2 Experiment Data

Two datasets were used in this study, where dataset 1 was recorded from our BCI system, and dataset 2 was Data set IIIa in BCI Competition III 2005 provided by BCI-Lab.

In dataset 1, EEG was recorded from two healthy male right-handed subjects aged 22 and 26 (P1, Y1) respectively. During the experiment, the subjects sat in a relaxing chair with armrests. The trial began with a fixation cross “+” appearing in the screen center. After 2 s' presentation of the fixation, a letter cue indicating the motor imagery for left hand, right hand and foot or tongue was presented. The subject was asked to perform the corresponding motor imagery. The experiments consisted of several runs (6 or 9 runs), each of which contained 40 trials and lasted about 9 minutes. All runs were conducted in one session with a 3–4 minutes break between them. In the experiment, the four movement tasks have the equal probability to appear (i.e., 25% for each task). The total number of trials was 240 for P1 and 360 for Y1. For the two subjects, the trials were split into a training set and an unlabeled test set. The recording was done by the Net Amps 200 systems with a 129-channel electrode cap (Electrical Geodesics Incorporated, USA), two channels for EOG and the other 127 for EEG. EEG was recorded with Cz as reference at sampling rate 250Hz, and the band-pass filter between 0.1 to 48Hz was applied to recordings.

The paradigm designed for dataset 2 was similar to that of dataset 1. The details can be found in [13]. Three subjects (K1, K6 and L1) participated in the experiment. The recording was made with a 64-channel EEG amplifier from Neuroscan, using the left mastoid as reference and the right mastoid as ground. The total number of trials was 360 for Subject K1 and 240 for the other two subjects (K6 and L1). The data were sampled at 250 Hz and filtered from 1 to 50 Hz. In our offline analysis, all data sets were down-sampled to 100Hz, and re-referenced to common average reference. The trials for each subject were also split into training and testing sets for performance evaluation.

### 3.3 Subject-Specific Feature Extraction

Event related synchronization and event related desynchronization (ERS/ERD) [14] could be observed over sensorimotor cortex during motor imagery tasks, and the experimental observation showed that particular mental tasks had related effects on the spatial distribution of EEG at μ (8–13Hz) and β (18–26Hz) rhythms. Further classification requires extraction of the rhythm related features from scalp EEG signals. In this study, in order to tackle the multi-class motor imaginary problems with binary classifiers, one-versus-one strategy [5] was adopted to change the multi-class problems to sub-binary problems. The final classification output is obtained by majoring voting for outputs of those sub-binary classifiers.

CSP has been proved to be an effective method to extract ERD/ERS related features from multi-channel EEG data of a two-motor imaginary task [15], [16]. The CSP extensions for multi-class problem have been shown in [14], [17]. Generally, the spatial filters were calculated individually for each subject, and such hyper-parameters of CSP as the frequency band, time section, optimal channel subset, μ and β band-pass filters could be semi-automatically estimated for each subject [18]. In our study, these hyperparameters were estimated in the following procedure.

#### 1) Activity regions for different patterns

**Figure 1:**
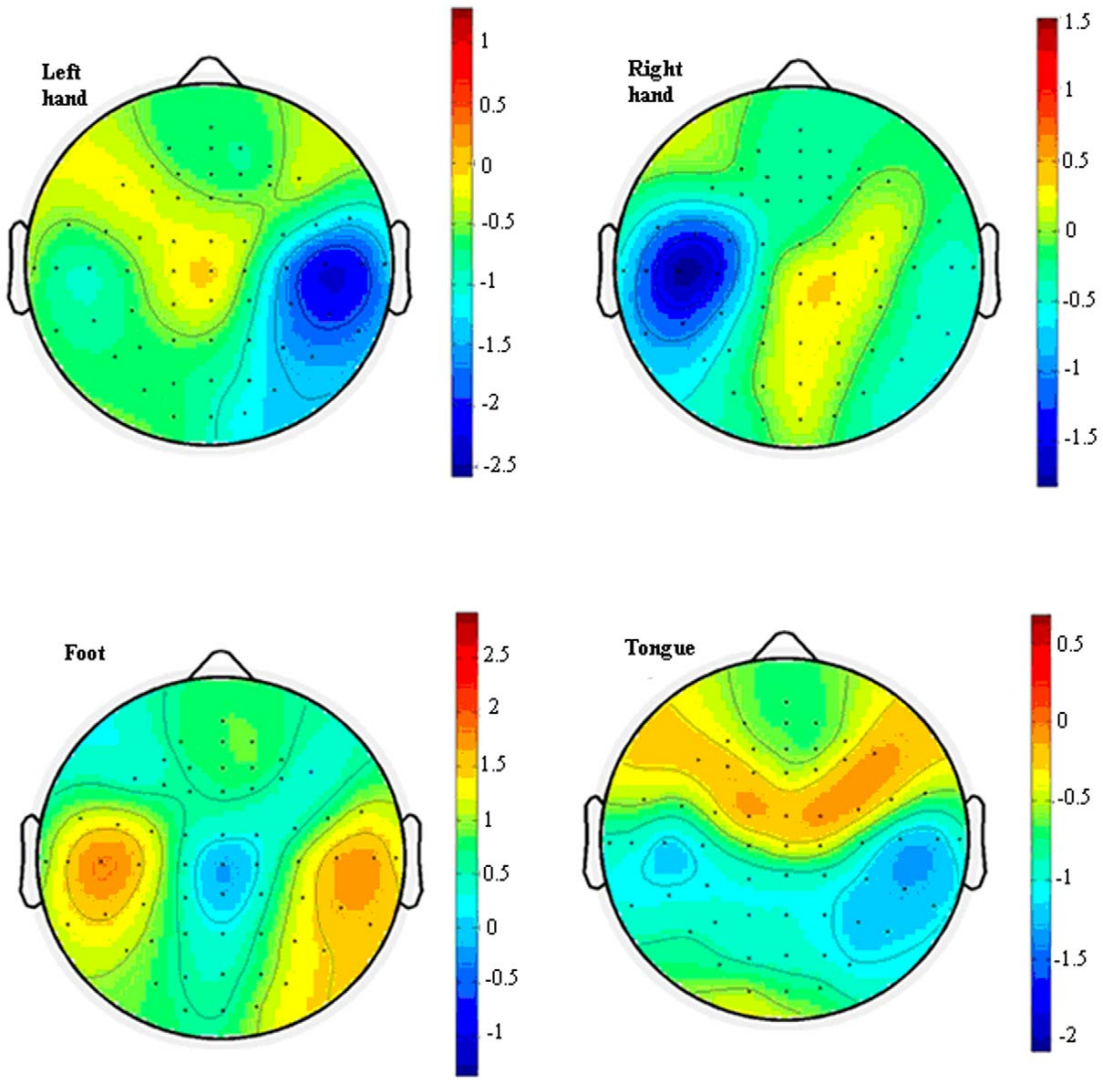
Activated regions of different imaging tasks of Subject K1 for the four motor imaging tasks, left hand, right hand, foot and tongue movements. The values (*r^2^*) in the figures are calculated according to equation (15). The optimal discrimination channels of different tasks were found to be located at C4 for left hand, C3 for right hand, Cz for foot and CP6 for tongue, respectively.

After the single trial log band power was estimated by spectrum estimation technique for each channel, the average band power during the motor imagery execution (Individual power) is calculated for each task based on each channel by averaging the task-correlated trials. The activity region of different patterns can be obtained by using Individual power minus Background power, where Background power is the average of band power over all the training trails of the four tasks. At last, the optimal discrimination channels of different tasks were selected by using below *r^2^*,
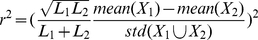
(15)where 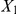 and 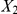 are the band power related features of certain class and the background power respectively, while 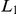 and 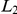 are the numbers of samples for different classes. Fig. 1 provides an example for Subject K1 that the optimal activity regions of different patterns were found to be located at C4 for left hand, C3 for right hand, Cz for foot and CP6 for tongue.

The related positions may vary across different subjects, but the similar pattern as that in Fig. 1 could be observed.

#### 2) Selection of optimal time interval

**Figure 2:**
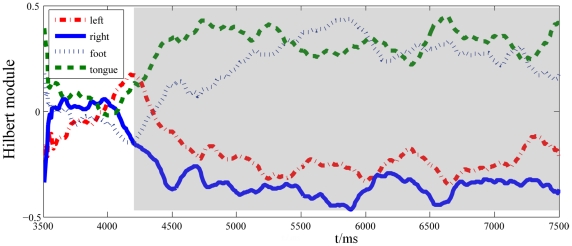
The average amplitude envelopes of the 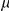 rhythm in time interval 3.5s—7.5s for subject K1. The curves are the Hilbert amplitude envelopes of the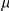 rhythm for the four motor imagery tasks, and the gray area indicates the optimal time window showing the obvious *r^2^* difference among the tasks.

The amplitude envelope of μ-rhythm for an optimal discrimination channel selected in the above step 1) was calculated by Hilbert transform and averaged over all training trials as the Background module. The amplitude envelopes during the execution of the four motor imagery tasks were calculated respectively as the Individual modules. The optimal time interval differentiating the four tasks was determined by the *r^2^*, which is defined in equation (15). Fig. 2 gives the optimal time interval 4.2s–7.5s for Subject K1.

The gray translucent rectangle marks the optimal time interval for the classification of the four different tasks.

#### 3) Band-pass filter used for μ and β rhythms

Though the ERD/ERS were observed for all the 5 subjects, the band of the μ and β rhythms were different among subjects. In this paper, the best band-pass filter was estimated according to both the best discrimination electrodes obtained from step 1) and the optimal time segments extracted from step 2) for each subject. In our study, 8–33Hz band filter is identified as a good choice for all the five subjects.

#### 4) Selection of channels for CSP

Selection of the discriminative channels was meaningful to lower the computation load for feature extraction and promote the stability of BCI. There are various techniques to select the channels [19]. In this study, we used the Greedy iterative to search for channel selection because it is easy to understand and implement. In each iteration of Greedy iterative algorithm, 4 channels randomly selected from the channel set were removed and the classification was performed using the remaining channels. The average classification result of a 2×5-fold cross-validation (CV) was used as the criteria for electrode selection. The iteration continued until all possible combinations of electrodes were tested. The best channel set was the channel combination that has the best classification accuracy in iterations.

#### 5) Selection of the CSP filters

**Figure 3:**
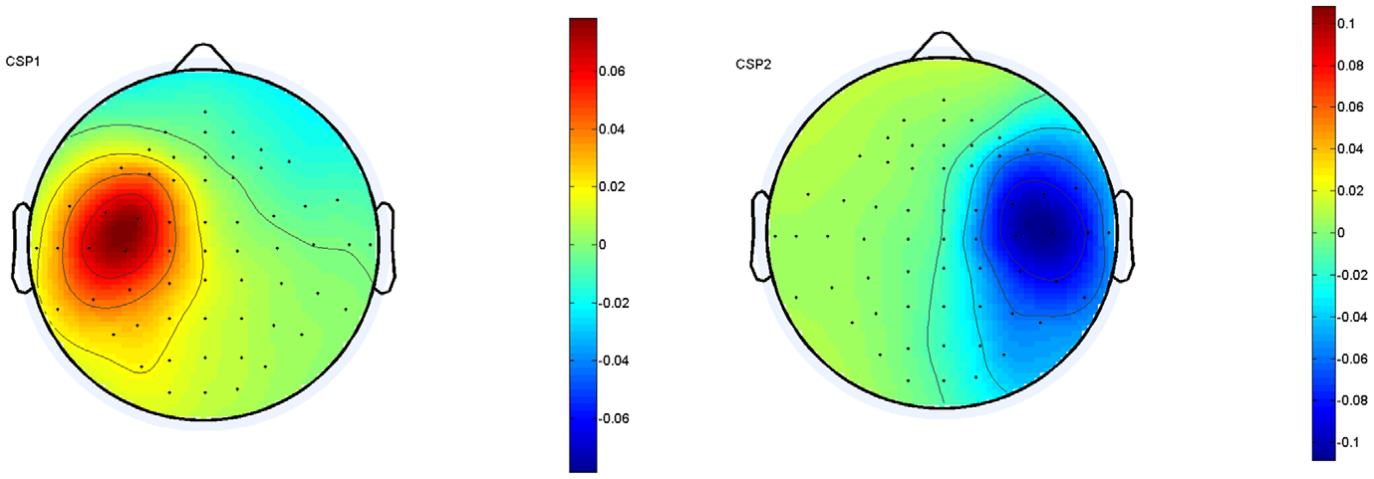
The scalp distributions of the CSP filters for subject K1 with performing right and left motor imagery. The two filters are defined by the largest and smallest eigenvalues in CSP decomposition, and the evoked ERDs for these two tasks can be reflected by the scalp distributions of the two CSP filters.

After electrodes were selected, CSP was used to extract the features for classification. In CSP, good contrasts were provided by the paired filters, which had high eigenvalue and low eigenvalue, respectively. In this study, the optimal number of CSP filters was chosen from the number set {2, 4, 6, 8} for each subject by a 2×5-fold cross-validation. Fig. 3 shows two dominant CSP filters for the right and left imaginary tasks for Subject K1.

In this paper, the log-variance of the CSP projected signals is used as features for classification.

In summary, the optimal filters for μ and β and the time section were determined from the selected best channel. Based on the time window and frequency band, the optimal channel subset was selected for a robust CSP implementation. Finally, the log-variance of CSP filtered time series was treated as feature for classification.

The above steps provided us with a fixed procedure to select the optimal parameters for different subjects. In this paper, we followed this fixed procedure to find the relatively optimal parameters and extract the rhythm related features for each subject.

### 3.4 Majority Voting

The one-versus-one decomposition transforms a *N*-class problem into *N(N−1)/2* binary classification problems, and the final classifier output for *N*-classes could be made by the voting of all the binary classifiers. Let 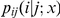 denote the probability of feature *x* belonging to class i when the classification is made between class *i* and class *j*. The below voting strategy could be used to obtain the probability of feature *x* belonging to class *i* with all binary classifiers (named as 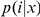) as,
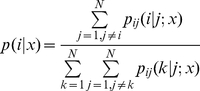
(16)


Based on the combination of classifiers, the input *x* is assigned into the class 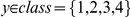 using the majority of votes,
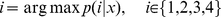
(17)


### 3.5 Kappa Coefficient

The kappa coefficient [20] is an evaluation criterion for unifying different number classification problems. In the *N* class problems, the proper performance measure of the classifier is described by its confusion matrix [20].If the *N* classes occur equally with probability of *1/N*, the relationship between kappa coefficient *k* and accuracy (*acc*) can be described as,
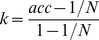
(18)where *acc* is the classification accuracy. In our work, there were an equal number of trials of each class in each session. Therefore, we took this simplified equation to evaluate the performance of classifiers.

In the algorithm realization, the parameters of BLDA and EBLDA (mean and covariance matrix) were not obtained by the time-consuming CV procedure in this study. Instead, we got these parameters by an iteration algorithm, which was constructed according to the probabilistic output model and allowed a quick and automatic parameters estimation with a few iterations as noted in Section *BLDA* [9].

## Results

### 1. Why and When do BLDA and EBLDA work?

**Figure 4:**
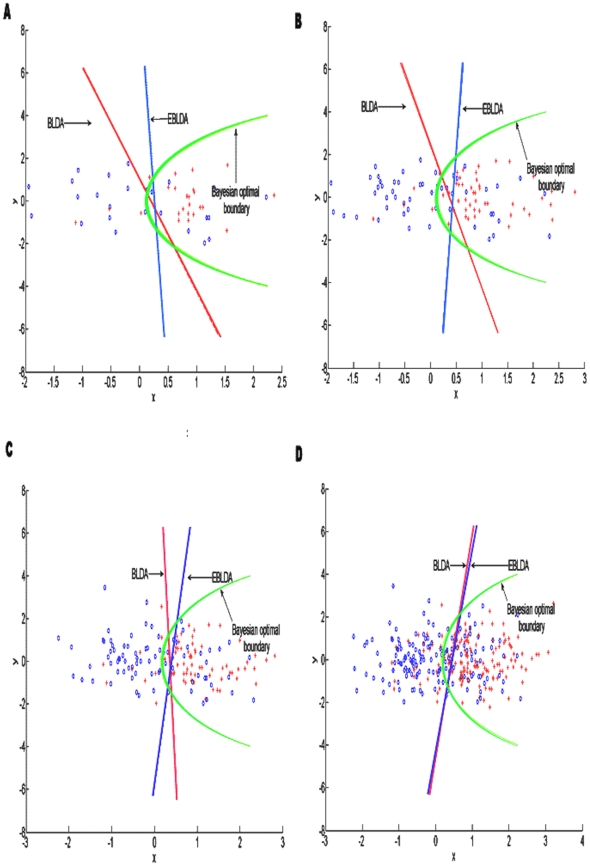
Decision boundaries and feature vector distribution of training sets derived from one of the 5×10-fold cross validation processes for simulated datasets. The green curve denotes the theoretical boundary, and the boundary curves for BLDA and EBLDA are in red and blue respectively. The blue circles and the red crosses represent the training samples of classes 1 and 2 respectively. (a) training set contains 50 samples; (b) training set has 100 samples; (c) training set has 150 samples; (d) training set has 200 samples. The size of the test set consists of 100 samples for (a), (b), (c) and (d), and EBLDA will select the samples with high probability from these 100 samples to enlarge the training set. The shift of decision boundary between BLDA and EBLDA was due to the combination of reliable samples with high probability in EBLDA.

As illuminated in section *the Simulated Data Sets*, the optimal decision boundaries can be determined by the distribution parameters of the simulated data (equation (14)). To observe the efficiency of EBLDA for the small training set, we increased the size of training sets from 20 to 200, and another 100 test samples were used to select high probabilistic samples. As for EBLDA, the samples from the original training sets combined with the selected samples were used to train the classifier. BLDA and EBLDA used the same test dataset, which contained 100 samples for performance evaluation. Fig. 4 reveals the change of boundaries between BLDA and EBLDA when the size of training set increased. Fig. 4 showed four cases where the initial training sample sizes were 50, 100,150 and 200 respectively. In Fig. 4, the blue circles and the red crosses represent the training samples of classes 1 and 2 respectively. The green solid lines are the Bayesian optimal decision boundaries of the two classes.

5×10-fold CV was adopted to obtain the average kappa coefficient for BLDA and EBLDA, where the 10-fold CV was repeated for 5 times. Meanwhile, the paired t-test was performed to investigate the difference between BLDA and EBLDA. Table 1 shows the mean Kappa and standard deviation of 5-fold cross validation with BLDA and EBLDA for the simulated dataset.

**Table 1:** The Kappa coefficients (%) for the simulated dataset.

Training size	20	50	80	100	150	200
**BLDA**	44.46±0.28	47.70±0.36	50.24±0.28	53.80±0.20	53.84±0.24	52.48±0.16
**EBLDA**	46.88±0.36	54.86±0.32	54.40±0.20	54.72±0.16	55.42±0.32	52.36±0.24
***p*** **-values** Δ	<0.01	<0.01	<0.01	<0.05	0.3333	0.5879

The calculation is based on a 5×10-fold cross validation with BLDA and EBLDA methods. The p-values of the paired t-test are in the fourth row.

Δ: P values are noted as <0.01(very significant), 0.05 (significant) or the true value for >0.05.

### 2. Experimental results

As for this dataset, the five steps described in III-C were firstly used to select the subject-specified parameters. As for the 4 motor imagery tasks with the one-versus-one CSP method, there were six individual binary-class groups in total, ({1,2}, {1,3}, {1,4}, {2,3}, {2,4}, {3,4}). We took the 2J CSPs filters that best discriminated each binary class problem to obtain the feature vector, where 2×5 fold CV was used to choose a suitable J in the range of 1∼6. The different 2J features vectors were obtained for each class group, and the final results were obtained by voting strategy. In our study, the optimal number of CSP filters was six for Subjects K6 and L1 and four for other subjects. BLDA and EBLDA were tested with a 5×10-fold cross–validation. To investigate the effect of training set size on classifier performance, the trials were split into three sets (i.e., the training set, the enlarging set for obtaining high probabilistic samples, the test set for evaluating the performance of BLDA and EBLDA). The size of training set ranged from 40 to 120 for all subjects. The size of the enlarging set was 60 for Subjects K6, L1 and P1 and 90 for Subjects K1 and Y1, and the size of test set equaled that of the enlarging set for each subject. In each CV procedure, EBLDA and BLDA had the same test set for performance evaluation and comparison. In this paper, as a model selection procedure, the values of the BLDA parameters (the mean and covariance matrixes) were automatically estimated by an iterative algorithm introduced in [11] according to the training set for different subjects. For the tested datasets, hyper-parameters optimization usually converged after eight to fifty iterations.

The classification performance for those five subjects when different approaches were used was listed in Table 2.

**Table 2:** The Kappa coefficients of 5×10-fold cross validations with BLDA and EBLDA for the experiment dataset.

Training size	methods	Subjects				
		K1	K6	L1	P1	Y1
40						
	**BLDA**	81.3±8.9	34.1±13.0	42.8±11.3	29.0±12.0	40.8±10.8
	**EBLDA**	82.6±9.0	32.9±11.5	42.8±10.8	29.0±11.0	41.1±10.4
	***p*** **-values**	0.1492	0.1167	0.9519	1	0.8089
60						
	**BLDA**	86.2±4.7	50.8±9.4	60.6±9.0	35.4±10.1	47.6±9.5
	**EBLDA**	87.9±4.1	52.3±9.6	61.1±9.5	33.8±11.6	47.1±8.5
	***p*** **-values**	<0.01	<0.05	0.1243	0.0874	0.7245
80						
	**BLDA**	87.9±4.3	53.2±9.0	63.4±9.4	38.9±8.5	52.8±8.2
	**EBLDA**	89.0±3.9	55.4±8.7	67.1±8.3	37.0±8.3	55.2±9.0
	***p*** **-values**	<0.01	<0.01	<0.01	<0.01	<0.01
100						
	**BLDA**	89.2±3.5	55.1±8.3	68.2±6.7	38.9±9.2	56.4±7.8
	**EBLDA**	90.4±3.2	57.3±8.4	69.5±6.3	39.1±8.7	57.1±8.0
	***p*** **-values**	<0.05	<0.01	<0.05	0.8032	<0.05
120						
	**BLDA**	89.6±3.4	56.5±8.2	69.6±6.1	40.5±7.7	57.3±5.6
	**EBLDA**	90.0±3.2	57.7±8.4	71.2±5.8	39.9±7.8	58.0±6.8
	***p*** **-values**	0.2314	<0.05	0.0657	0.0785	<0.05

The feature vectors are obtained by one-versus-one CSP methods. The performance of BLDA and EBLDA classification methods are estimated with different training sizes.

**Figure 5:**
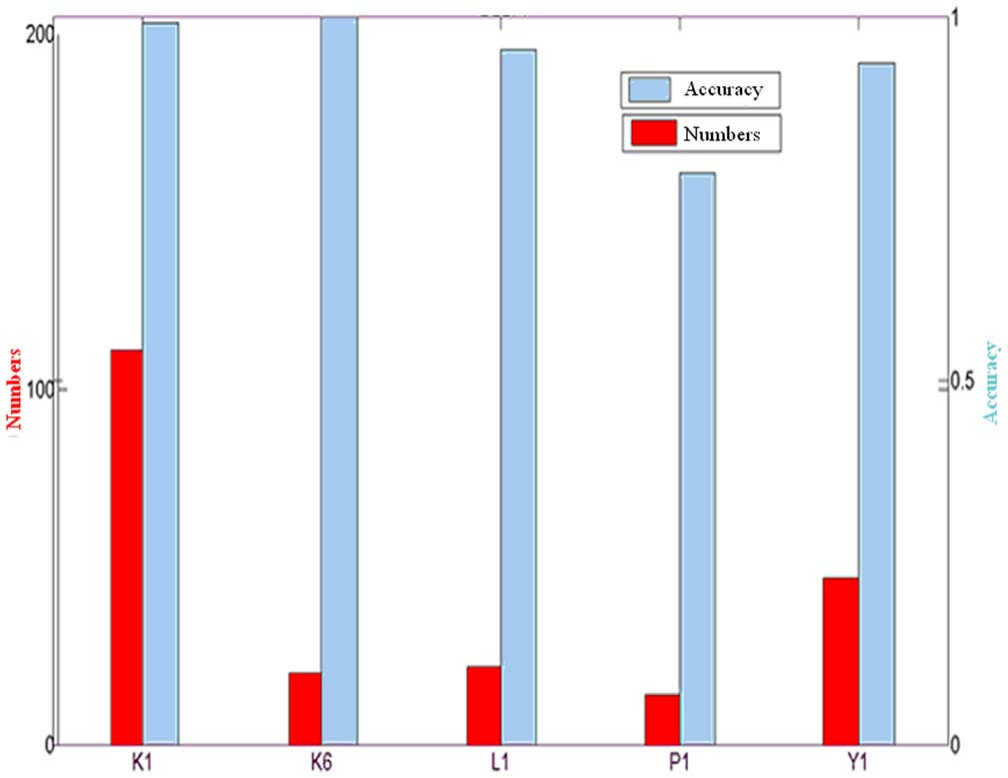
The selection of reliable samples and the corresponding accuracy of BLDA. The number of selected reliable samples and the corresponding classification accuracy when probability threshold is kept to be 0.90 for the 5 subjects. The plot is derived from one of 5×10-fold cross validation for the five subjects. The red bar using the scale of the left axis represents the number of trials selected for expanding training set; the gray one using the scale of the right axis is the classification accuracy when the reliable samples are used for classifier training.

**Figure 6:**
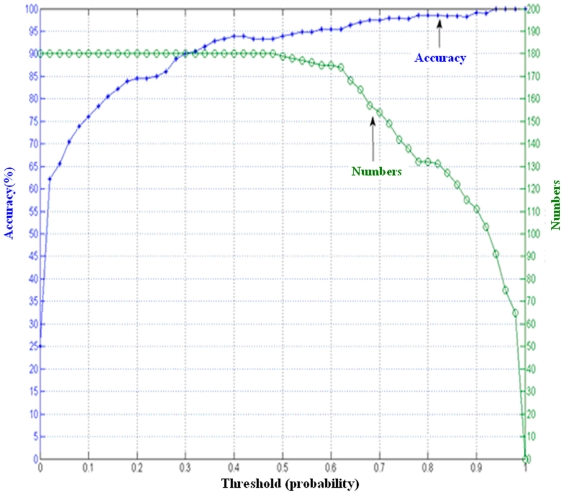
The curves of probability threshold vs accuracy (blue) and probability threshold vs number of selected reliable samples (green) for subject K1. The blue curve uses the scale of the left axis, and the green curve uses the scale of the right axis.

The number of the test samples selected for enlarging training set was determined by the probability threshold, which was used for reliable sample selection. The one–versus-one decomposition strategy transformed the four-class problem into six binary subtasks, ({1,2}, {1,3}, {1,4}, {2,3}, {2,4}, {3,4}), where three classifiers were related to one task. Fig. 5 illustrated the number of high probabilistic test samples in the test samples and the corresponding accuracies achieved by combining those high probabilistic test samples for classification in EBLDA. In Fig. 5, the confident threshold is 0.90 for all subjects, and those test trials having probabilities above 0.90 will be added to the training set for classifier re-training. Fig. 6 shows the curves of probability threshold vs accuracy (blue) and probability threshold vs number of selected reliable samples (green) for subject K1.

## Discussion

### 1. EBLDA vs BLDA

Fig. 4 reveals that the classification boundary of BLDA shifted to the tangent direction of Bayesian optimal interface when the training sets were expanded by adding the reliable test samples that had high probability. The angle between BLDA to EBLDA became small when the size of training set increased, suggesting that the boundaries of BLDA and EBLDA were more similar to each other. Furthermore, the final decision boundaries of these two classification methods had a high degree of overlap when the training set had enough samples. Table 1 reveals that the EBLDA method produced a significantly better kappa than BLDA with a training size of 20 to 100 (t-test, *p*<0.05). The results suggest that high probabilistic test samples can refine the BLDA boundary and EBLDA can obtain a more stable result using information from the reliable test samples. As a linear classifier, based on BLDA [9], EBLDA can solve the overfitting problem better than other nonlinear counterpart like LDA, MD, when a limited sample size is available. Furthermore, EBLDA can also relearn information from new samples, which can produce a better boundary for normal distributions especially when a small sample set is used. The kappa coefficients shown in Table 1 also show that the expansion of the training set with reliable test samples improves the classifier performance, where more obvious improvement can be observed for small training set. Generally, EBLDA has better performance using the information in the additional high probabilistic test samples, and it could be a refined version of BLDA.

### 2. Multi-class problem and majority voting

Unlike the two-class problem, a multi-class problem may have unclassifiable region where a sample may have an equal chance to be classified into a few classes when a 0/1 voting is adopted. As for the real dataset containing four tasks, we adopted probability majority voting, which could automatically remove uncertainty.

### 3. Selection of threshold and reliable test samples

Fig. 6 demonstrates that with larger threshold, the accuracy increased and the selected samples will be reduced. Besides, as shown in Fig. 5, the size of training samples for Subjects K1 and Y1 is 180 and 120 for other subjects (K6, L1, P1). The rest of the trials are treated as test samples. When 0.9 is used as the confident threshold, the number of test samples added to training set is about 1/3∼1/2 of the size of the test sets for most of the subjects. According to Fig. 6, all test samples are added to the training set if we set a probabilistic threshold smaller than 0.5 for Subjects K1, but the accuracy may be small. If we choose a high threshold such as 0.75, the classification accuracy will be higher than 0.9 for K1. This fact suggests that if the test samples with high probability could be selected for classifier training, the classification accuracy could be substantially improved. On the other hand, the unreliable information introduced by the trials with small probability will distort the classifier performance. In this study, 0.9 is a good threshold for Subjects K1 and Y1, 0.85 is good for subjects K6, L1, and 0.8 is suitable for subject P1. This threshold has to be estimated for each individual subject and the selection criteria is to include as many high probability samples as possible while high accuracy could be guaranteed at the same time.

### 4. EBLDA for individual subjects

As Table 2 shows, EBLDA generated better results than BLDA for all subjects except P1. The averaged classification Kappa coefficients of the five subjects ranged from 0.586 to 0.657. When the training size increased, the kappa coefficient of EBLDA did not fluctuate as much as that obtained by BLDA, especially for subjects with high classification accuracy. For Subject P1 with low classification accuracy, the classification result of EBLDA was even worse than that of BLDA when the training set was expanded. One possible explanation is that when the training set was expanded with misclassified samples, the classifier would be distorted by the unreliable information from those misclassified samples. However, for subjects with high classification kappa coefficients (i.e., K1, L1 and Y1), the performance of EBLDA was better than that of BLDA, suggesting that EBLDA can learn useful information from the correctly classified test samples to improve the classification accuracy, especially for subjects with high kappa coefficients.

### 5. Conclusion and prospect

The results confirmed that EBLDA could achieve substantial improvement over the traditional BLDA algorithm, especially when the size of training set is small. Since the unlabeled test samples added to the training set are strictly selected by the probability threshold, EBLDA could produce a more creditable result than BLDA. Some previous studies have confirmed that BLDA can get more reliable results for the multi-class classification compared to the traditional classifier like LDA, MD, SVM, and accordingly EBLDA can have superior performance to those traditional counterparts according to the performance relationship between BLDA and EBLDA revealed in this paper.

In summary, BLDA is robust to noise in the training data [9], and can capture information from test samples without much human intervention. Since Bayesian approach of prediction could take the posteriori uncertainty of the parameters into account, it could produce a more accurate estimation of the uncertainty in predictions, especially when the training data do not have enough information for a precise estimation of the model parameters. Based on BLDA, EBLDA can efficiently use the additional information from test samples for classifier calibration. EBLDA also has other properties, which are very important for practice of BCI. First, EBLDA can obtain probabilistic output, which can be used to reject trials that cannot be classified with certainly. Therefore, it could help alleviate the negative effect of wrong decisions [9]. Furthermore, probabilistic output can be used for continuous control with high classification results. Finally, the hyperparameters of BLDA can be estimated quickly, which may satisfy the demand for real-time BCI communication.
